# Disentangling Environmental Variability and Mining Disturbance: A Five-year Assessment of Mammal Community Responses to Open-pit Expansion in a Semi-arid Landscape

**DOI:** 10.1007/s00267-026-02549-3

**Published:** 2026-07-07

**Authors:** Ruan W. Higgs, Gert Nicolaas Smit, Francois Deacon

**Affiliations:** Department of Animal, Wildlife and Grassland Sciences, University of the Free State, Bloemfontein, 9300 South Africa

**Keywords:** open-pit mining, biodiversity, camera traps, species richness, conservation management, South Africa

## Abstract

Open-pit mining is a major driver of habitat transformation, yet separating mining impacts from natural environmental variability remains challenging in semi-arid ecosystems. We evaluated mammal detection patterns over five years (2020–2024) in relation to mining expansion, rainfall, vegetation productivity, water availability, and habitat type in the Northern Cape Province, South Africa. Wildlife monitoring was conducted using 52 fixed camera-trap stations distributed across approximately 43,000 ha, generating 14,360 trap-nights. Detection activity varied with rainfall and herbaceous productivity, reflecting expected resource-driven dynamics. However, three nocturnal species, aardwolf (*Proteles cristatus*), Cape fox (*Vulpes chama*), and Cape porcupine (*Hystrix africaeaustralis*), showed consistent declines associated with mining expansion into wild olive shrubland. Detection rates were negatively correlated with the extent of mining disturbance (r = –0.55 to –0.72).

Because detection probability was not explicitly modelled, results are interpreted as activity-based associations rather than measures of abundance. Nevertheless, the consistency of observed spatial and temporal patterns suggests that mining-related habitat modification may influence wildlife activity beyond background climatic variability. These findings highlight the value of integrating long-term camera-trap monitoring with spatial disturbance mapping to support biodiversity management in semi-arid mining landscapes.

## Highlights

- Five years of camera-trap monitoring (14,360 trap-nights) were used to evaluate mammal responses to open-pit mining expansion in a semi-arid South African landscape.
- Mammal detection activity varied with rainfall and herbaceous productivity, demonstrating the importance of environmental variability in shaping wildlife patterns.
- Mining expansion increased the disturbed footprint by approximately 30% and resulted in encroachment into wild olive shrubland habitat.
- Aardwolf (*Proteles cristatus*), Cape fox (*Vulpes chama*), and Cape porcupine (*Hystrix africaeaustralis*) showed consistent negative associations with mining expansion.
- Wildlife responses were habitat-specific, indicating that vegetation heterogeneity mediates the ecological effects of industrial disturbance.
- Integrating long-term biodiversity monitoring with spatial disturbance mapping provides practical guidance for environmental impact assessment and adaptive mine management.

## Introduction

Habitat loss and fragmentation from human activities are leading drivers of biodiversity decline globally (Haddad et al. 2015). In semi-arid ecosystems, wildlife populations are strongly influenced by environmental variability, particularly rainfall-driven fluctuations in vegetation productivity (Gandiwa et al. 2016). At the same time, vegetation structure, interspecific interactions, climatic conditions, and anthropogenic disturbance interact to shape community composition and spatial use (Brown et al. 2001; Tews et al. 2004). Distinguishing natural variability from anthropogenic drivers is therefore essential for environmental impact assessment and adaptive management (Čepič et al. 2022).

Open-pit mining represents an intensive form of land-use transformation involving vegetation removal, soil disturbance, infrastructure development, and continuous industrial activity (Cristescu et al. 2016; Sonter et al. 2018; Boldy et al. 2021). These processes can alter habitat structure, modify resource availability, and introduce sensory disturbance (noise, light, traffic), potentially influencing wildlife behaviour and spatial distribution.

A previous study conducted within this system documented 44 mammal species over a five-year camera-trap survey and reported reduced species richness at sites located nearer to mining activity (Higgs and Deacon 2024). Those findings demonstrated spatial heterogeneity in species richness but did not evaluate species-specific responses or integrate environmental variability with mining expansion. Patterns of lower richness near mining activity suggested possible displacement or reduced site use, but the relative roles of climatic variability, habitat characteristics, and spatial mining expansion were not examined.

The present study builds on that dataset using a species-specific analytical framework. We evaluate how detection rates of nine focal mammal species vary in relation to (i) rainfall and vegetation productivity, (ii) water availability, (iii) habitat differences among vegetation units, and (iv) spatial and temporal expansion of mining activities. The objective is to identify consistent spatial and temporal patterns that may inform management decisions under ongoing land-use change.

## Materials and Methods

### Study Area

The study was conducted in and around a large open-pit iron ore mine near Postmasburg, Northern Cape Province, South Africa, spanning approximately 43,000 ha across 17 properties. The region lies within the Griqualand West Centre of Endemism (Frisby 2016). Eight vegetation units were delineated based on plant community composition and geomorphology.

Mining infrastructure includes active pits, waste rock dumps, haul roads, processing facilities, and associated lighting installations. Operations occur continuously, including nocturnal activity. Artificial lighting is present at pit edges, processing areas, and along main haul roads. Vehicle traffic includes heavy haul trucks operating throughout the day and night. No quantitative noise measurements were collected as part of this study; however, large-scale open-pit operations typically generate continuous mechanical and blasting-related noise (Pantelić et al. 2023). The area does not contain perennial rivers. Surface water is limited to artificial water points (boreholes and dams) established for livestock and mining operations.

#### Data Collection

Camera-Trap Design and Deployment:

Wildlife data were collected using 52 passive infrared camera traps deployed between January 2020 and December 2024. Cameras were fixed in permanent locations throughout the study period and did not rotate among sites. Each camera station constituted a sampling unit.

Cameras were distributed across the eight vegetation units (see Appendix 1) as follows: Black Thorn Shrubland (*n* = 8), Camphor Bush Panveld (*n* = 8), Dwarf Karroid Shrubland (*n* = 6), Rhigozum Grassland (*n* = 6), Senegalia melifera Shrubland (*n* = 6), Wild Olive Shrubland (*n* = 7), Wolhaarkop Sandveld (*n* = 6), Mining-disturbed footprint (*n* = 5).

Cameras were placed to represent habitat variation and spatial gradients relative to mining activity. Mean inter-camera distance was approximately 1.25 km (minimum 0.56 km), reducing spatial dependence among stations. Cameras were mounted at approximately 40–50 cm above ground level on metal stakes or trees, oriented along animal movement pathways where feasible but not baited. A total of 14,360 trap-nights were accumulated. Independent detection events were defined as consecutive photographs of the same species separated by ≥30 min. Detection rate (events per 100 trap-nights) was used as an index of relative activity.

### Vegetation Surveys and Productivity

**Fig. 1:**
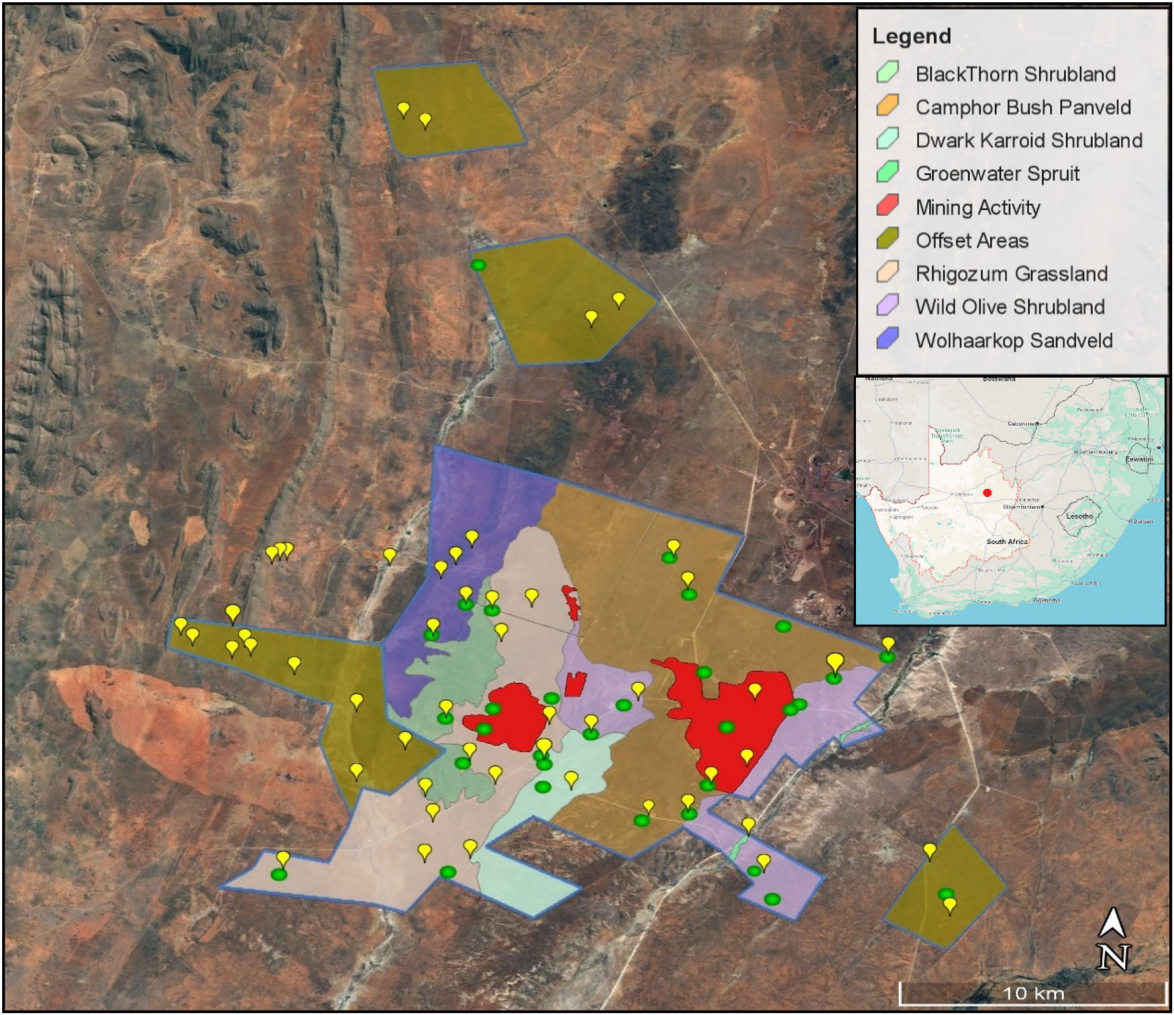
Study area showing vegetation units and monitoring locations. The map illustrates the eight vegetation units identified across the study area. Yellow markers indicate fixed camera-trap stations, green markers indicate vegetation monitoring plots, and red polygons represent the active mining footprint. The inset map shows the location of the study area within South Africa

To track habitat condition, vegetation monitoring plots were surveyed annually as part of the mine’s environmental management plan. We analysed data from 26 fixed vegetation plots (100 m × 25 m each) distributed across the eight vegetation units shown in Fig. 1. Surveys conducted from 2010 to 2024 recorded plant species composition and ground cover. Veld condition was assessed for each plot using the ecological index method of Vorster (1982), following Heard et al. (1986). Above-ground herbaceous biomass, expressed as dry matter production (DMP), was estimated by clipping and weighing vegetation in sample quadrats (Smit 2010). Woody biomass was estimated using the BECVOL model (Smit 2014), which calculates browse volume and mass from shrub dimensions.

For each year, herbaceous DMP was averaged across plots within each vegetation unit to provide an annual index of vegetation productivity. The present analysis focused on interannual trends in productivity over the 2020–2024 period corresponding to the camera-trap survey. Because herbaceous biomass estimates were derived from an established long-term monitoring programme, the same method was retained to preserve comparability across years. We acknowledge that destructive harvesting approaches are less suitable for repeated monitoring than newer non-destructive methods, and future work should incorporate complementary remote-sensing approaches such as NDVI, UAV imagery, or related spectral methods where feasible.

Mining expansion was quantified separately using Landsat 8 imagery (30 m spatial resolution) together with mine planning GIS shapefiles for 2020, 2022, and 2024. The active disturbance footprint, including pits and waste-rock dumps, was digitised and its extent calculated in hectares. The proportion of each vegetation unit affected by mining was then computed for each of the three time points.

### Climatic and Environmental Data

Meteorological data were obtained from the mining company’s on-site weather station. We compiled total monthly rainfall (mm), mean daily temperature (°C), and mean daily relative humidity (%) for each month from 2020 to 2024. These records enabled us to characterise seasonal and annual climate variability during the study. In the latter two years (2023–2024), the region experienced notably below-average rainfall relative to 2020–2022, alongside a slight downward trend in mean humidity. We used these data to explore correlations between climate variables and wildlife detection rates.

We also noted the presence or absence of artificial water points (boreholes or dams) within ~200 m of each camera trap as a categorical variable. Surface water availability can concentrate wildlife in arid environments (Redfern et al., 2003). We therefore tested whether cameras near water sources had higher species richness or detection rates, given that placing camera traps near water can increase detections in dry regions (Edwards et al., 2016).

### Data Analysis

All statistical analyses were conducted in R version 4.2.0 using the packages stats, dplyr, and ggplot2. Relationships between wildlife detection metrics and environmental variables were evaluated using Pearson’s correlation coefficients (Sedgwick 2012). For each of the nine focal species, correlations were calculated between mean detection rate and annual rainfall, vegetation dry matter production, distance from the active mine, presence of water, and mammalian species richness recorded at each camera station. Pearson correlations were also used to examine associations among focal-species detection rates in order to identify patterns of positive or negative co-occurrence.

Because cameras were maintained at fixed locations throughout the study, potential spatial dependence was reduced by the spacing of camera stations across vegetation units and by analysing annual mean detection rates at the station level. Detection probability was not modelled explicitly because the sampling design did not include repeat detection occasions structured for occupancy-based approaches (MacKenzie and Royle 2005). Detection rates should therefore be interpreted as indices of relative activity or site use rather than direct measures of abundance. Moran’s I tests conducted on residuals indicated no significant spatial autocorrelation.

Correlation strength was interpreted using conventional thresholds, with |r | > 0.5 considered strong, 0.3–0.5 moderate, and < 0.3 weak. Statistical significance was assessed using two-tailed t-tests at α = 0.05. Associations with *p*-values between 0.05 and 0.10 were retained as marginal trends given the exploratory nature of the analysis.

Mining expansion was quantified using annual mine planning data together with Landsat 8 imagery (30 m spatial resolution) for 2020, 2022, and 2024. The footprint of active pits and waste rock dumps was mapped for each time point, and the total area directly disturbed by mining was calculated. From these spatial layers, the proportion of each vegetation unit affected by mining was quantified, and correlations were then examined between expansion into wild olive shrubland and detection rates of the three species that showed the strongest declines.

Given the absence of standardised camera-occasion detection histories, hierarchical modelling approaches such as generalised linear mixed models, occupancy models, or N-mixture models could not be applied consistently across the dataset. Accordingly, all results are interpreted as associations rather than causal relationships.

## Results

### Rainfall, Vegetation Productivity, and Overall Wildlife Trends

**Fig. 2:**
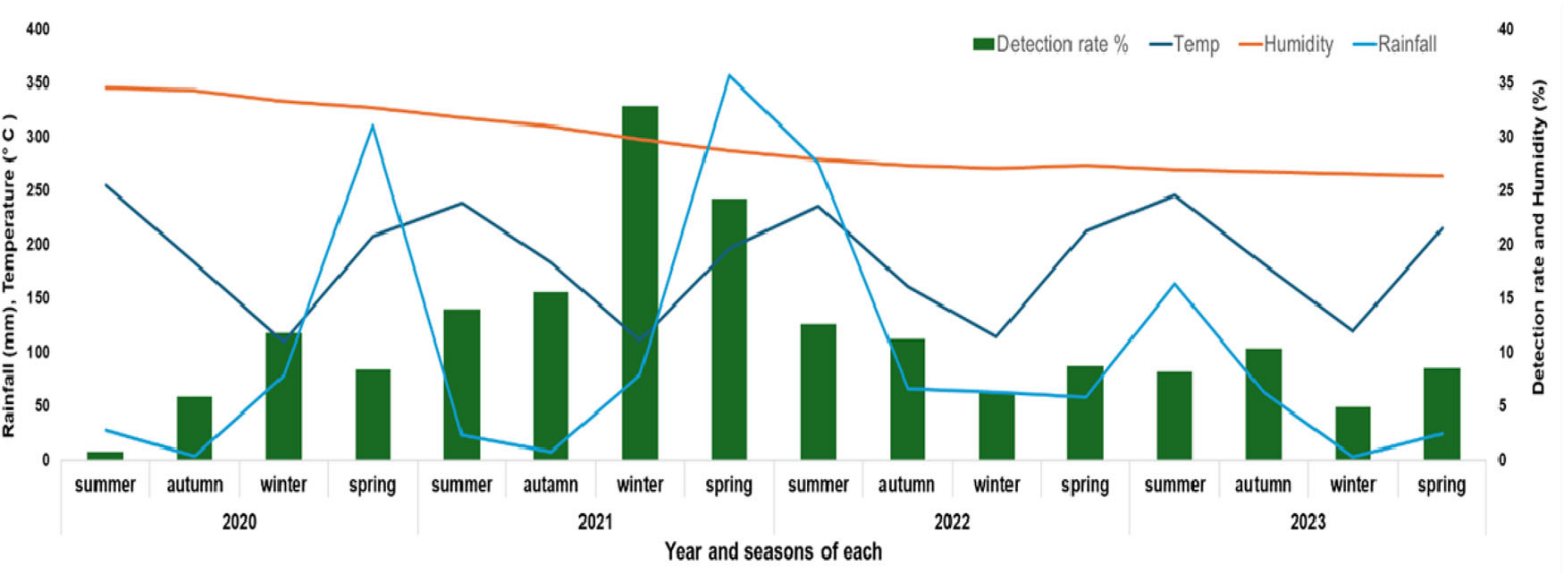
Seasonal climatic conditions and mammal detection activity during the study period. The figure illustrates seasonal variation in mean temperature (°C), relative humidity (%), rainfall (mm), and average mammal detection rates from 2020 to 2024. Green bars represent average mammal detection rates, while the coloured lines represent temperature, humidity, and rainfall as indicated in the legend

Total annual rainfall declined over the study period, from approximately 380 mm in 2020 to 210 mm in 2024. This reduction in precipitation was accompanied by a corresponding decline in herbaceous vegetation productivity across the study area. All vegetation units showed an increase in mean dry matter production (DMP) between 2020 and 2021 following higher rainfall, after which DMP declined sharply in 2022 and remained low through 2024 (Fig. 2). An analysis of variance indicated a significant decline in plant biomass over time (F = 3.37, *p* = 0.044). Temporal patterns in DMP closely followed interannual rainfall variability (Fig. 2).

**Fig. 3:**
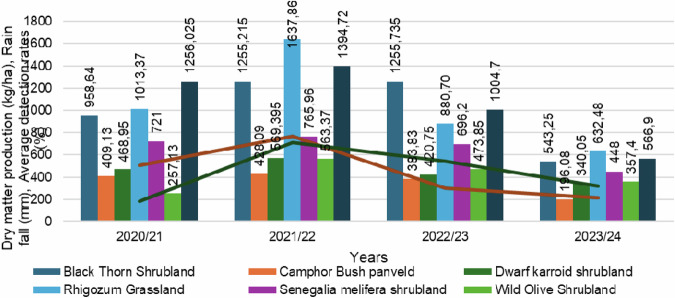
Annual vegetation productivity and rainfall across vegetation units. The figure shows annual dry matter production (kg ha⁻¹) for the seven vegetation units from 2020/21 to 2023/24 together with annual rainfall. Coloured bars represent the individual vegetation units, while the brown line represents annual rainfall (mm)

Across the same period, the total number of mammal species detected annually remained relatively stable, ranging between approximately 40 and 42 species. However, temporal variation in overall detection rates was observed. Higher detection activity occurred during 2021, coinciding with higher rainfall and vegetation productivity, whereas lower detection rates were recorded during the drier conditions of 2023–2024 (Fig. 3). Correlation analysis indicated a weak positive relationship between annual rainfall and overall mammal detection rate (r = 0.31), although this relationship was not statistically significant (*p* = 0.226; Table 1). In contrast, mean herbaceous DMP showed a moderate positive correlation with overall detection rates (r = 0.64), approaching statistical significance (*p* = 0.051; Table 1).

**Table 1:** Pearson correlation coefficients between mammal detection rates and environmental factors (rainfall and vegetation productivity). The correlation with mean dry matter production of vegetation was marginally non-significant (*p* = 0.051)

Factor	Pearson Correlation (*r*)	*t*-Statistic	*p*-Value
Rainfall	0.311	1.254	0.226
Dry Matter Production	0.644	2.090	0.051*

### Species-Specific Responses to Environmental Factors

The nine focal species exhibited variable relationships with environmental factors (Table 2). Across vegetation types, correlation coefficients were generally weak ( | r | < 0.3). Negative correlations were observed for aardwolf (*Proteles cristatus*) and Cape fox (*Vulpes chama*) with wild olive shrubland extent, although these relationships were not statistically significant (*p* > 0.10).

**Table 2:** Pearson correlation coefficients (r) between focal mammal species detection rates and environmental variables, including vegetation type, water presence, local species richness, and distance from mining activity

Species	Scientific Name	Vegetation type	Water Presence	Species Richness	Distance from mining activity
**Aardwolf**	*Proteles cristatus*	–0.019	–0.026	0.356	–0.110
**African Wild Cat**	*Felis lybica*	0.051	–0.061	0.279	0.071
**Black-Backed Jackal**	*Canis mesomelas*	–0.196	0.075	0.500*	–0.161
**Cape fox**	*Vulpes chama*	0.210	0.281	0.160	–0.093
**Cape porcupine**	*Hystrix africaeaustralis*	0.218	0.434	0.420	0.429
**Caracal**	*Caracal caracal*	–0.055	0.355	0.237	0.019
**Common Duiker**	*Sylvicapra grimmia*	0.012	0.436	0.407	0.175
**Greater Kudu**	*Tragelaphus strepsiceros*	–0.158	0.468*	0.370	–0.116
**Scrub Hare**	*Lepus saxatilis*	–0.157	–0.041	0.327	–0.166

Single asterisk (*) indicates *p* < 0.10; double asterisk (**) indicates *p* < 0.05.

Water availability showed stronger associations for several herbivorous and omnivorous species. Detection rates of greater kudu (*Tragelaphus strepsiceros*), common duiker (*Sylvicapra grimmia*), and Cape porcupine (*Hystrix africaeaustralis*) were moderately positively correlated with proximity to water sources (r = 0.47, 0.44, and 0.43, respectively; Table 2). The relationship for kudu approached statistical significance (*p* = 0.096), while the other relationships were not significant at α = 0.05.

Local species richness at camera sites was moderately positively correlated with detection rates of black-backed jackal (*Canis mesomelas*) (r = 0.50) and Cape porcupine (r = 0.42; Table 2). The correlation for jackal approached significance (p = 0.081), while the porcupine relationship remained non-significant at the 95% confidence level. Distance from active mining areas showed generally weak relationships across species, with the exception of Cape porcupine. Detection rates for this species increased moderately with increasing distance from mining activity (r = 0.43), with marginal statistical support (p ≈ 0.10).

### Inter-Species Associations

Correlation analysis among focal species revealed several significant positive associations (Table 3). Detection rates of African wildcat (*Felis lybica*) were positively correlated with those of black-backed jackal (r = 0.67, p = 0.048) and scrub hare (*Lepus saxatilis*) (r = 0.72, p = 0.048). Similarly, scrub hare detections were positively correlated with black-backed jackal (r = 0.67, p = 0.049). Aardwolf detections were moderately positively correlated with black-backed jackal (r = 0.61), although this relationship was only marginally significant (p = 0.055). No significant negative correlations were observed among focal species.

**Table 3:** Correlation matrix for detection rates among nine focal mammal species

Species	Aardwolf	African Wild Cat	Black-Backed Jackal	Cape fox	Caracal	Common Duiker	Greater kudu	Porcupine	Scrub Hare
Aardwolf	—	0.356*	0.610*	-0.110	-0.055	0.012	-0.158	0.218	-0.157
African Wild Cat	0.356*	—	0.670**	0.210	0.051	0.279	-0.061	0.218	0.720**
Black-Backed Jackal	0.610*	0.670**	—	-0.196	0.075	0.500*	-0.161	0.420	0.670**
Cape fox	-0.110	0.210	-0.196	—	0.355*	0.160	0.281	0.429	-0.093
Caracal	-0.055	0.051	0.075	0.355*	—	0.237	0.355*	0.019	0.327
Common Duiker	0.012	0.279	0.500*	0.160	0.237	—	0.407*	0.420**	0.407
Greater kudu	-0.158	-0.061	-0.161	0.281	0.355*	0.407*	—	0.370	0.327
Porcupine	0.218	0.218	0.420	0.429	0.019	0.420**	0.370	—	-0.041
Scrub Hare	-0.157	0.720**	0.670**	-0.093	0.327	0.407	0.327	-0.041	—

Values are Pearson correlation coefficients (*r*). Single asterisks (*) denote marginal significance at α = 0.10; double asterisks (**) denote significance at α = 0.05. All significant correlations were positive; no significant negative correlations were found among these species.

### Mining Expansion and Impacts on Wildlife

**Fig. 4:**
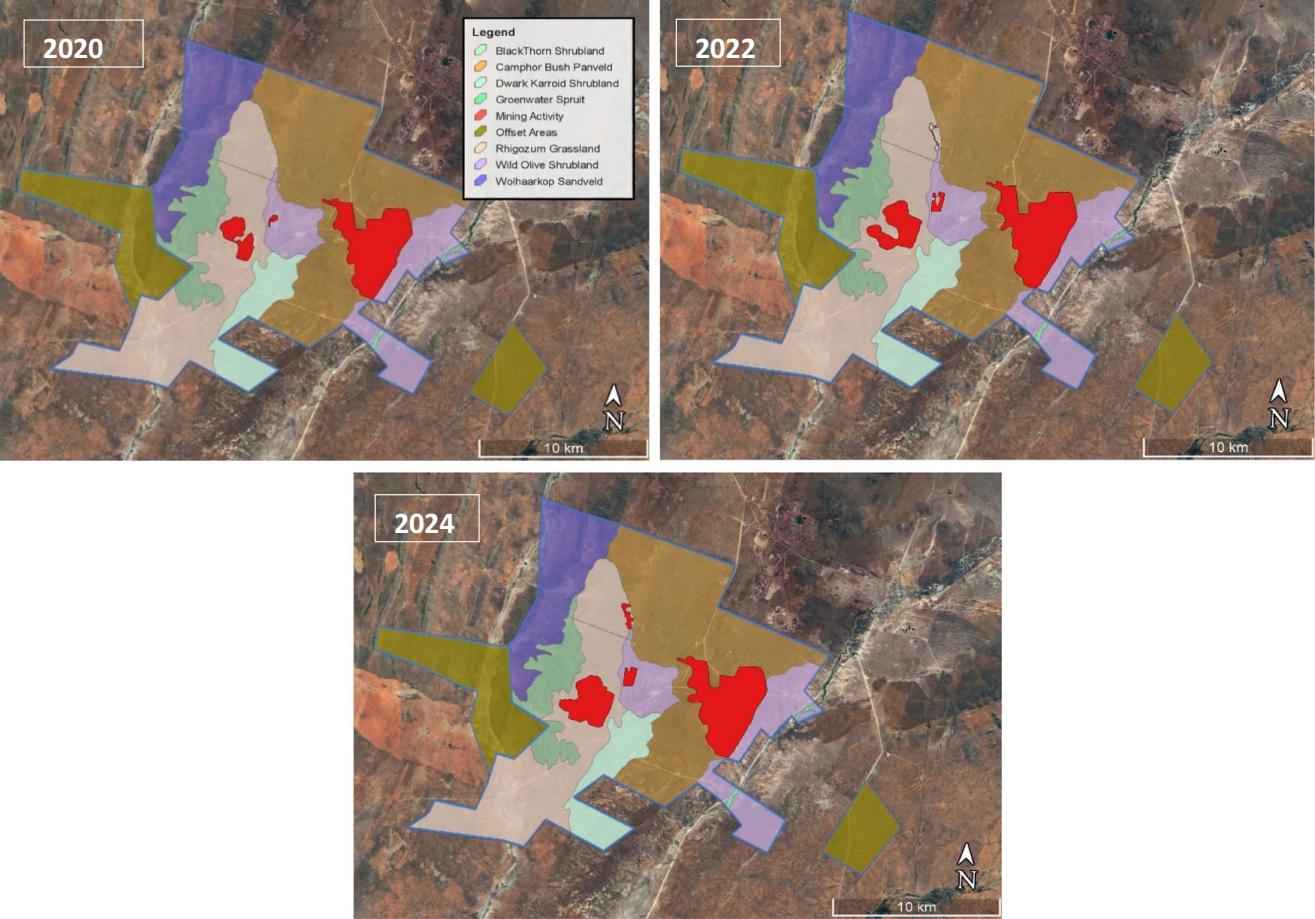
Expansion of the mining footprint between 2020 and 2024. **a** Mining footprint and vegetation units in 2020, **b** mining footprint in 2022, and **c** mining footprint in 2024. Red polygons represent active mining areas, while the remaining colours denote the diff erent vegetation units, illustrating the progressive expansion of mining into adjacent habitats over the study period

During the five-year study, mining operations expanded substantially in area. In 2020, the active mining footprint covered approximately 2025 ha (6.9% of the main property). By 2024, this had increased to approximately 2661 ha (8.9% of the property), representing a 30% increase in surface area directly disturbed. The expansion occurred primarily in a northwesterly direction from the original pit, extending into adjacent wild olive shrubland habitat (Fig. 4). Specifically, the proportion of the wild olive shrubland vegetation unit affected by mining increased from 0.42% in 2020 to 1.12% by 2024, resulting in the loss of a measurable portion of this habitat.

Concurrently, detection rates of the aardwolf, Cape fox, and Cape porcupine declined within and near wild olive shrubland areas. These species already exhibited comparatively lower detection rates at camera stations located close to active mining areas during the early years of the study, and their detections decreased further over time as the mining footprint expanded. Correlation analysis indicated negative relationships between the extent of mining intrusion into wild olive shrubland and detection rates of aardwolf (r = –0.55) and Cape fox (r = –0.55) (Table 4). These relationships were marginally significant at the 90% confidence level (p = 0.066 for both species). The Cape porcupine exhibited a stronger negative association (r = –0.72), which was statistically significant at the 95% confidence level (*p* = 0.048).

**Table 4:** Correlation between the expansion of the mine (intrusion into wild olive shrubland habitat) and detection rates of three affected species. Negative Pearson *r* values indicate that as mining area increased, detection rates decreased

Species	Pearson correlation (*r*)	*t*-Statistic	*p*-Value
Aardwolf	–0.554	–1.958	0.066*
Cape fox	–0.554	–1.958	0.066*
Cape porcupine	–0.719	–2.120	0.048**

* *p* < 0.10; ** *p* < 0.05.

**Fig. 5:**
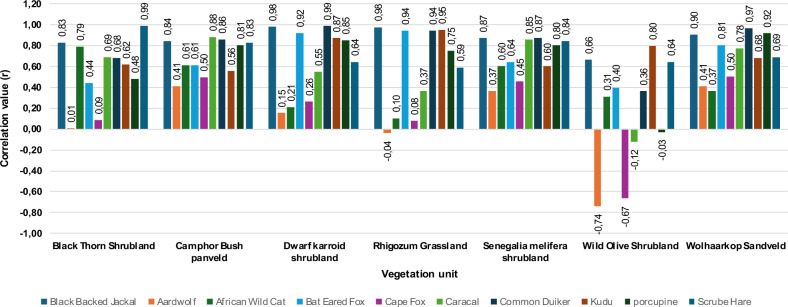
Correlations between focal mammal species and vegetation units. The figure illustrates Pearson correlation coefficients (*r*) between detection rates of the nine focal mammal species and the seven vegetation units. Positive values indicate positive associations, whereas negative values indicate negative associations between species detections and vegetation units. Different coloured bars represent the individual mammal species as indicated in the legend

Additional patterns were observed within the wild olive shrubland vegetation unit. Independent of mining expansion, aardwolf and Cape fox detection rates were lower in this vegetation unit relative to others across the study area (Fig. 5), although these relationships were not statistically significant when considered in isolation. The caracal also showed a negative association with this habitat type, although this relationship was weak and not statistically significant (Table 2). By 2024, camera trap stations located within or immediately adjacent to active mining areas, predominantly situated within wild olive shrubland, recorded very low detection frequencies for aardwolf and Cape fox. In contrast, these species continued to be detected at comparatively higher frequencies in shrubland areas located further from mining activity.

Other species exhibited less consistent spatial patterns in relation to mining expansion. The black-backed jackal and common duiker were detected across a wide range of distances from mining activity, with no clear directional trends in relation to mine proximity. The aardvark (*Orycteropus afer*), although not included among the primary focal species due to low detection frequency, exhibited a strong negative correlation with mining expansion (*r* = –0.97). However, this relationship was not statistically significant (*p* = 0.323), likely reflecting the limited sample size for this species.

## Discussion

**Fig. 6:**
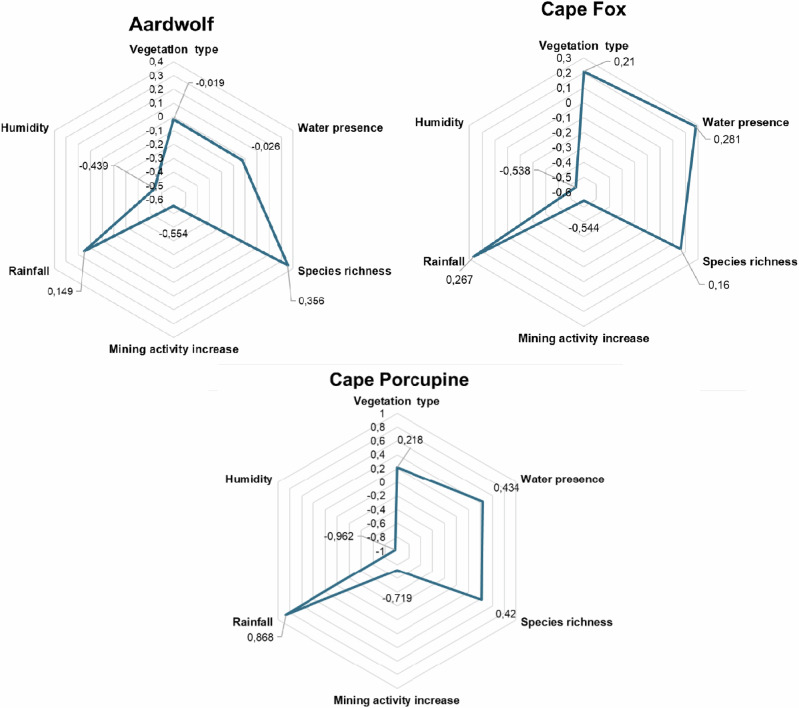
Correlation profiles of three mammal species in relation to environmental variables and mining expansion. **a** Aardwolf (*Proteles cristatus*), **b** Cape fox (*Vulpes chama*), and **c** Cape porcupine (*Hystrix africaeaustralis*). Radar plots illustrate Pearson correlation coefficients between detection rates and environmental variables, including vegetation type, water presence, species richness, rainfall, humidity, and mining activity increase. Positive values indicate positive associations, whereas negative values indicate negative associations with the respective environmental variable

**Fig. 7:**
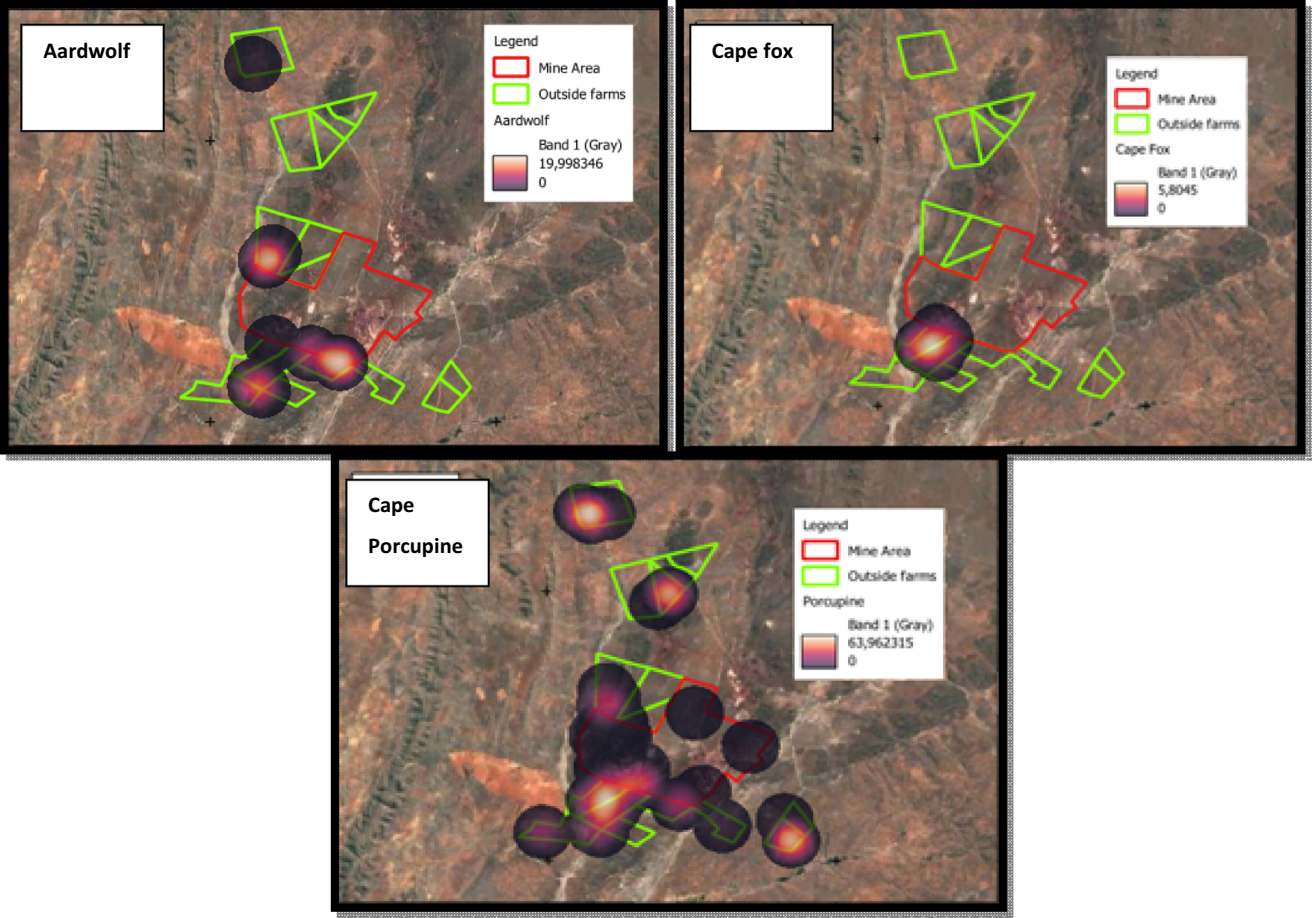
Spatial distribution of detection activity for three nocturnal mammal species across the study area. **a** Aardwolf (*Proteles cristatus*), **b** Cape fox (*Vulpes chama*), and **c** Cape porcupine (*Hystrix africaeaustralis*). Heat maps show the relative spatial intensity of camera-trap detections across the monitoring network. Warmer colours indicate higher detection intensity, while cooler colours indicate lower detection intensity. Red polygons represent the mining footprint and green polygons indicate surrounding properties outside the mine area

**Fig. 8:**
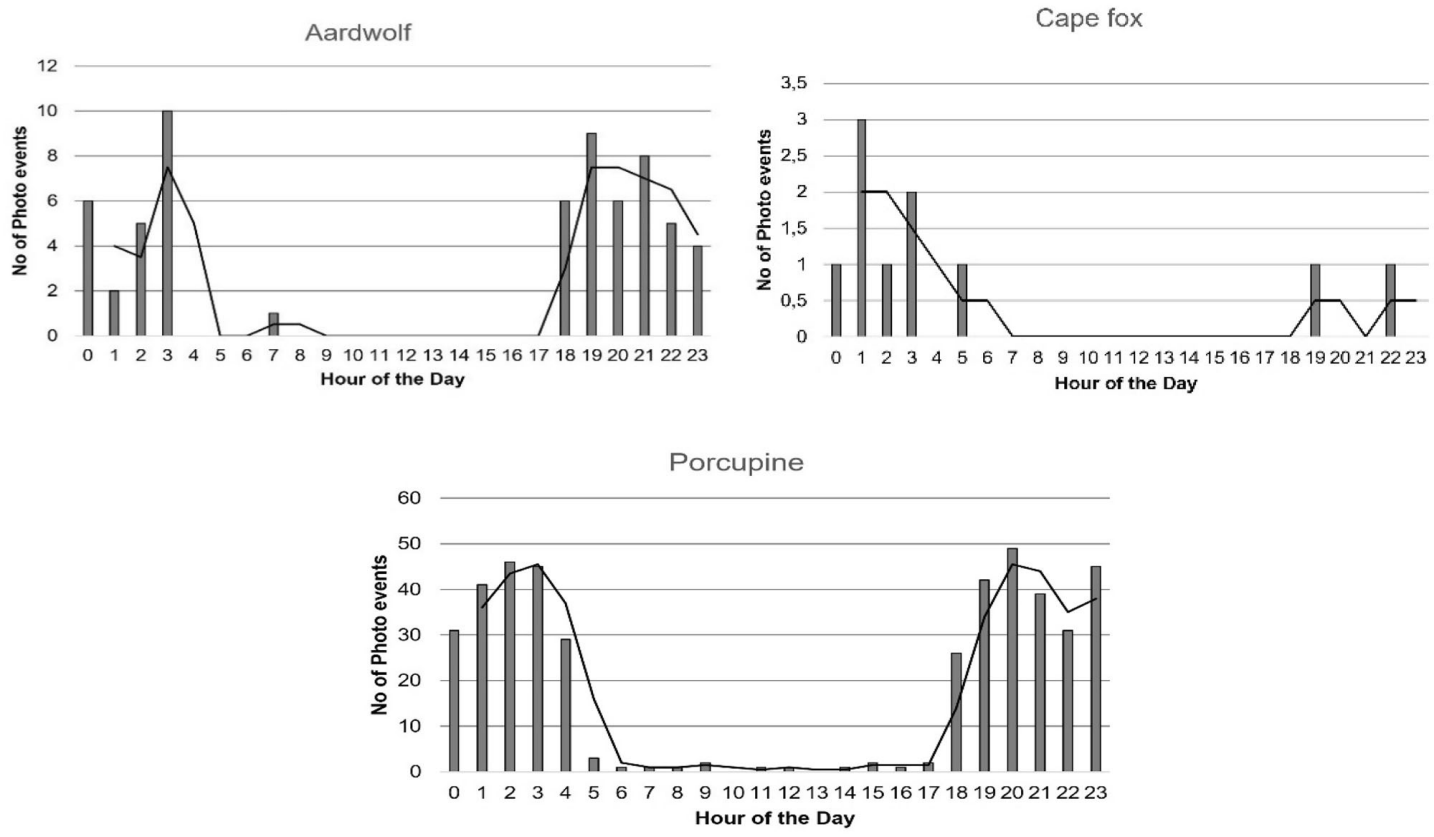
Diel activity patterns of three nocturnal mammal species recorded by camera traps. **a** Aardwolf (*Proteles cristatus*), **b** Cape fox (*Vulpes chama*), and **c** Cape porcupine (*Hystrix africaeaustralis*). Histograms show the number of independent detection events recorded for each hour of the day (0–23 h), with the overlaid line illustrating the temporal activity trend derived from the hourly detections

**Fig. 9:**
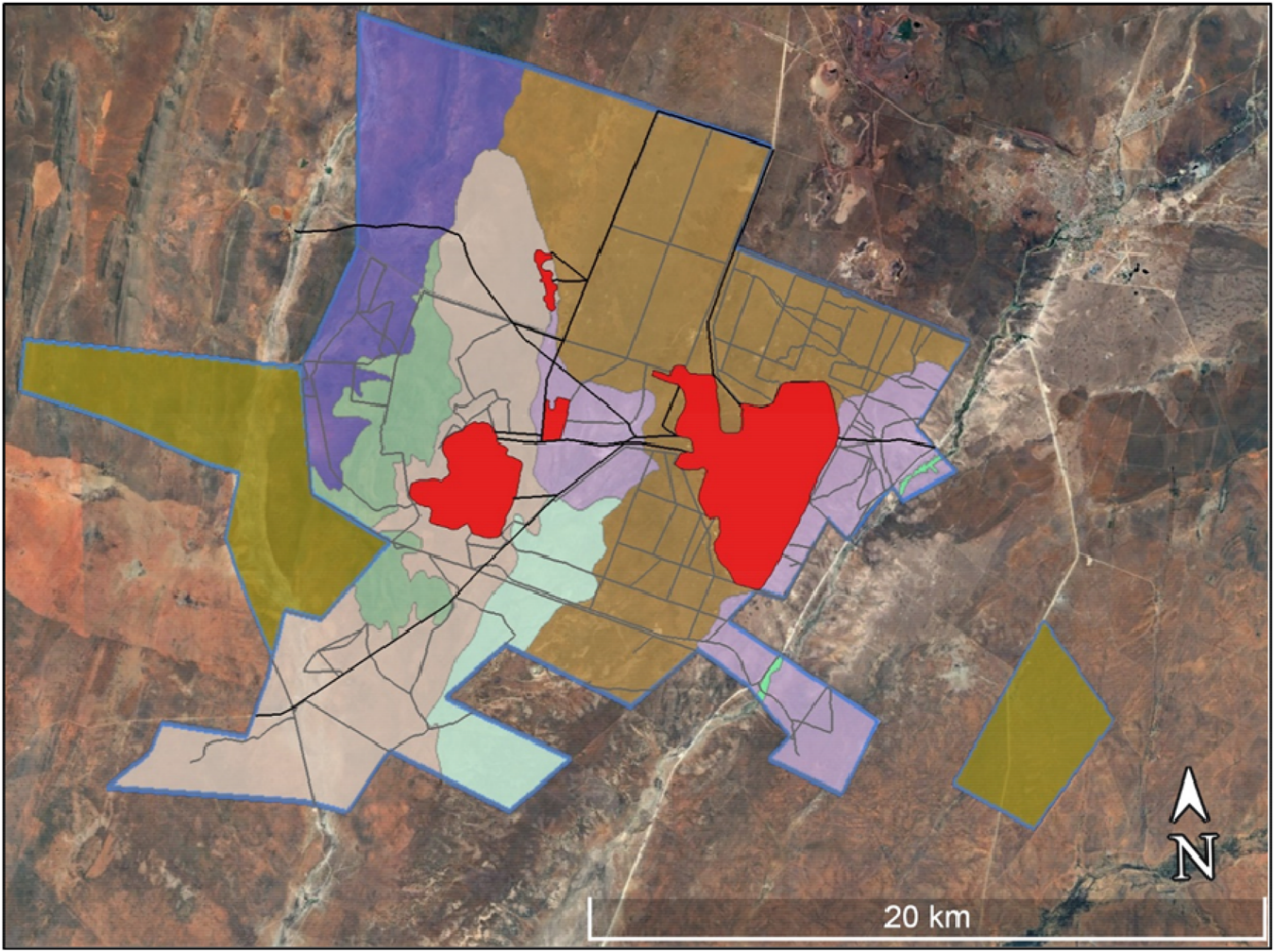
Study area showing vegetation units, mining footprint, and road network. The map illustrates the distribution of vegetation units across the study area together with the active mining footprint (red), the main access roads, and secondary roads used within the mining landscape. The figure provides spatial context for the camera-trap monitoring network and the observed wildlife detection patterns

This study integrates five years of camera-trap monitoring with environmental and spatial disturbance gradients to evaluate mammal responses within a semi-arid mining landscape on the Ghaap Plateau. By combining long-term wildlife detections with rainfall, herbaceous productivity, water availability, vegetation units, and spatial mining expansion, the study provides a more detailed ecological interpretation than a simple comparison of species richness or mine proximity alone. The principal contribution of the dataset lies in its ability to examine how mammal detection patterns vary through time under changing climatic conditions, while also showing how those patterns are distributed across a heterogeneous vegetation mosaic undergoing progressive mining disturbance. In this respect, the study contributes not only to mining ecology, but also to understanding how habitat-specific responses emerge within a semi-arid plateau system of high ecological sensitivity Figs. 6–9.

The temporal dimension of the study is particularly important. The monitoring period captured both relatively wet conditions during 2020–2021 and a pronounced dry phase during 2023–2024, enabling evaluation of mammal responses across contrasting environmental states. Increased rainfall during the early phase coincided with higher herbaceous dry matter production and elevated overall detection activity, which is consistent with ecological theory describing resource-pulse dynamics in arid and semi-arid systems (Brown et al. 2001; Gandiwa et al. 2016). In many African savanna systems, variation in vegetation productivity affects herbivore distribution and can, in turn, influence broader faunal activity patterns through changing resource availability (Redfern et al. 2003). The present results are consistent with that broader pattern. Conversely, reduced rainfall and declining herbaceous productivity during later years coincided with lower overall detection rates, suggesting that reduced resource availability likely contributed to shifts in wildlife activity or site use across the landscape.

At the same time, climatic variability alone does not appear sufficient to explain the full pattern observed in the study area. Annual species richness remained relatively stable across the monitoring period, while some of the clearest changes in detections were spatially concentrated near the expanding mining footprint. This distinction is important because it suggests that the mammal community was not simply responding to drought or declining productivity in a uniform manner. Rather, temporal environmental variability and spatial mining expansion appear to have operated simultaneously, with rainfall and vegetation productivity contributing most clearly to the temporal signal, and mining expansion contributing most clearly to the spatial redistribution of detections for selected species. This distinction strengthens the interpretation of the study because it shows that broad environmental fluctuation and localised industrial disturbance did not produce identical ecological patterns.

The most pronounced mining-associated responses were observed for *Proteles cristatus*, *Vulpes chama*, and *Hystrix africaeaustralis*, all of which declined in association with mining expansion into wild olive shrubland. These responses are notable because they were not expressed uniformly across all focal species, nor were they equally distributed across all vegetation units. Instead, the clearest negative associations were concentrated in one structurally distinct habitat type. This pattern suggests that the biological expression of mining disturbance was filtered through the vegetation mosaic of the Ghaap Plateau. The results therefore indicate that mining expansion was not merely associated with a general decline in detections near disturbed areas, but with a more specific reorganisation of mammal activity where industrial expansion intersected a habitat unit likely to provide cover, shelter, movement pathways, and feeding opportunities for nocturnal and mesopredatory species.

This vegetation-context finding represents one of the more novel aspects of the study. Broad correlations between focal species and vegetation types were generally weak when considered independently, which could be interpreted superficially as evidence that vegetation units were of limited ecological importance. However, the mining results show a more nuanced pattern. Vegetation units did not need to show uniformly strong standalone associations to be ecologically important. Rather, they structured where mining expansion translated into the clearest biological response. Wild olive shrubland emerged as the vegetation unit in which habitat loss, fragmentation, and disturbance exposure appeared to converge most strongly. This is ecologically important for the Ghaap Plateau because it demonstrates that vegetation heterogeneity provides the spatial template through which mammal responses to disturbance become visible. The study therefore contributes a habitat-explicit perspective that is often lacking in generalised mining-impact assessments.

The Ghaap Plateau context further strengthens the importance of these findings. This landscape is not a homogeneous arid surface, but a mosaic of vegetation units that differ in structure, composition, and likely ecological function. Within such a system, mammal detections cannot be interpreted fully without considering how vegetation units mediate access to cover, prey resources, water, and disturbance exposure. The present study shows that the interaction between animal detections and vegetation units is not trivial background variation, but an important component of how wildlife responses are expressed under mining pressure. From a regional ecological perspective, this provides rare long-term evidence from a semi-arid plateau system where industrial disturbance, vegetation heterogeneity, and climatic variability can be evaluated within a single monitoring framework.

Several non-exclusive mechanisms may contribute to the observed associations. Habitat loss and structural modification are likely central. Expansion of mining infrastructure into wild olive shrubland removed and fragmented a portion of a habitat unit that appears to differ structurally from more open surrounding vegetation. Such change may reduce shelter availability, alter prey or food distribution, disrupt movement routes, and increase exposure to disturbance. Increased road networks and vehicle activity may further contribute to functional fragmentation by creating barriers to movement or intensifying local disturbance. In addition, industrial operations introduce continuous human presence, mechanical activity, artificial lighting, and other disturbance pathways that have been shown elsewhere to affect wildlife behaviour and space use (Bowles 1995; Rasmussen et al. 2009; Slabbekoorn et al. 2018; Shier et al. 2020). In the present study, however, these mechanisms should remain plausible explanatory pathways rather than confirmed drivers, because they were not measured directly.

The discussion of noise and light therefore needs to remain appropriately restrained. Continuous industrial noise can alter animal communication and induce behavioural stress, especially in species that rely strongly on acoustic cues (Bowles 1995; Slabbekoorn et al. 2018). Artificial light at night can disrupt circadian rhythms and modify nocturnal foraging behaviour or habitat use (Shier et al. 2020). These processes are biologically plausible in the current mining context, particularly for nocturnal species such as *P. cristatus*, *V. chama*, and *H. africaeaustralis*. However, because neither noise levels nor light intensity were quantified directly in this study, these mechanisms should be interpreted as informed hypotheses that are consistent with the observed spatial patterns, rather than as demonstrated causes.

Not all species responded similarly to mining expansion, and this variation is itself informative. *Canis mesomelas* and *Sylvicapra grimmia* were detected across a wider range of distances from mining activity, suggesting greater ecological flexibility or tolerance of disturbed conditions. Similarly, moderate associations between water availability and detections of *Tragelaphus strepsiceros*, *Sylvicapra grimmia*, and *H. africaeaustralis* indicate that local resource distribution continued to influence species patterns alongside disturbance gradients. This heterogeneity of response highlights the importance of considering species-specific ecology and functional traits when assessing disturbance effects. It also strengthens the management value of the study, because it shows that mining-associated change is selective and habitat-mediated rather than uniform across the mammal assemblage.

The inter-species results provide an additional layer of ecological insight. Positive associations among *Felis lybica*, *Canis mesomelas*, and *Lepus saxatilis*, together with the marginal positive association between *P. cristatus* and *C. mesomelas*, indicate that the camera-trap landscape was not structured primarily by strong exclusionary interactions at the spatial scale sampled. Instead, the significant associations suggest shared use of particular sites or habitat patches, possibly because those sites retained favourable combinations of vegetation cover, prey availability, or lower disturbance exposure. This is a useful extension of the study because it moves the interpretation beyond single-species responses and begins to show how disturbance, habitat structure, and species assemblage patterns intersect. In the context of the Ghaap Plateau, where long-term wildlife datasets remain limited, this represents a meaningful ecological contribution.

The novelty of the study also lies in its integration of long-term monitoring with spatially explicit disturbance and habitat information. Many studies have shown that mining can alter biodiversity patterns, reduce habitat area, and affect ecological processes (Cristescu et al. 2016; Sonter et al. 2018; Boldy et al. 2021). What this study adds is a finer-grained, species-level perspective from an understudied semi-arid African plateau in which mammal detections are linked not only to mine expansion, but also to rainfall variation, vegetation productivity, water availability, and vegetation-unit context. The findings therefore help bridge the gap between broad international syntheses on mining and biodiversity and the practical requirements of environmental monitoring and impact assessment. In particular, the study shows how a fixed long-term camera-trap network can identify which species appear most responsive, where in the habitat mosaic those responses are concentrated, and how disturbance signals compare with background environmental variability.

Several limitations should be recognised. Detection rates derived from camera-trap data represent indices of activity or site use rather than direct measures of abundance. Without repeated standardised detection histories, it was not possible to apply occupancy-based models that explicitly account for imperfect detection (MacKenzie and Royle 2005). Likewise, although camera stations were distributed across vegetation units, the study was not designed as a fully spatially explicit modelling framework for formal treatment of spatial autocorrelation beyond the structure of the fixed camera network. Disturbance mechanisms such as noise intensity, light levels, traffic volume, and direct habitat-use metrics were also not quantified independently. The results should therefore be interpreted as ecologically meaningful spatial and temporal associations rather than definitive evidence of local population decline or direct causal effect.

These limitations, however, do not diminish the value of the observed patterns. The consistency of the responses across years, camera locations, vegetation units, and focal species indicates that the relationships identified are unlikely to be trivial. The integration of wildlife detections with vegetation and disturbance gradients provides a practical framework for identifying areas of concern and improving environmental management in dynamic industrial landscapes. In this regard, the study offers an applied example of how biodiversity monitoring can move beyond simple species inventories toward more spatially informative ecological interpretation.

From a management perspective, the findings emphasise the importance of maintaining habitat heterogeneity within active mining landscapes. Wild olive shrubland appears to be of particular ecological importance because it was the vegetation unit most clearly associated with the mining-related responses of several nocturnal species. Limiting further encroachment into such habitats, reducing fragmentation where feasible, and prioritising rehabilitation of structurally important vegetation units may help reduce impacts on wildlife. In addition, management interventions aimed at reducing unnecessary nocturnal disturbance, improving lighting design, and limiting avoidable disturbance along key habitat interfaces may contribute to improved coexistence between mining operations and wildlife, although the effectiveness of such measures requires direct evaluation.

This study also underscores the value of long-term ecological monitoring. Camera-trap datasets maintained over extended periods provide critical insights into temporal variability and can reveal emerging trends that may not be evident in short-term surveys. When integrated with vegetation mapping and disturbance data, such datasets become especially powerful for informing adaptive management. On the Ghaap Plateau, where ecological sensitivity, vegetation heterogeneity, and mining pressure occur together, integrated monitoring of this kind is likely to be far more informative than treating wildlife, vegetation, and industrial expansion as separate assessment domains.

Overall, the patterns identified here support the view that wildlife responses to mining on the Ghaap Plateau are selective, habitat-mediated, and superimposed on strong background climatic variability. The study therefore contributes not only to understanding mammal responses in a mining landscape, but also to the broader ecological interpretation of how species, vegetation units, and industrial expansion interact in semi-arid plateau systems.

## Conclusions

This study provides a five-year assessment of mammal detection patterns within a semi-arid mining landscape on the Ghaap Plateau by integrating long-term camera-trap monitoring with rainfall, vegetation productivity, water availability, vegetation-unit context, and spatial mine expansion. The findings show that mammal detections were structured by both temporal environmental variability and localised mining-related landscape change. Detection activity varied through time in association with rainfall and herbaceous productivity, whereas the clearest spatial changes were concentrated in areas where mining expanded into wild olive shrubland.

The results therefore indicate that wildlife responses in this system were not expressed as a uniform mine-versus-non-mine pattern across all species. Instead, responses were selective, habitat-mediated, and superimposed on strong background climatic variability. In particular, *Proteles cristatus*, *Vulpes chama*, and *Hystrix africaeaustralis* showed consistent negative associations with mine expansion into wild olive shrubland, suggesting that this vegetation unit may represent an important structural habitat for disturbance-sensitive nocturnal mammals. These findings emphasise that vegetation heterogeneity on the Ghaap Plateau is central to understanding how mining disturbance is experienced by wildlife, and that habitat context should be treated as a core component of biodiversity assessment rather than as background landscape description.

An important contribution of the study is its demonstration that long-term fixed camera-trap datasets can be used not only to document species presence, but also to identify how species-specific detection patterns shift through time and across vegetation units in response to expanding industrial disturbance. In this respect, the study adds a spatially explicit, habitat-based perspective to the broader literature on mining and biodiversity, and provides management-relevant insight from an ecologically sensitive semi-arid plateau landscape that remains underrepresented in international scholarship.

The findings also have direct implications for environmental management. The concentration of mining-associated responses within wild olive shrubland suggests that structurally distinct vegetation units should receive particular attention in mine planning, biodiversity monitoring, and rehabilitation. Limiting further encroachment into such habitats, reducing fragmentation where feasible, and incorporating habitat-specific mitigation measures into environmental management plans may improve biodiversity outcomes. Measures aimed at reducing unnecessary nocturnal disturbance, including lighting management and minimisation of avoidable disturbance along key habitat interfaces, may also be beneficial, although their effectiveness requires direct evaluation.

Several limitations remain important. Detection rates derived from camera traps represent indices of activity or site use rather than direct measures of abundance. Detection probability was not modelled explicitly, and disturbance mechanisms such as noise, light intensity, and traffic volume were not quantified independently. The conclusions are therefore necessarily limited to association-based interpretation, and the observed patterns should not be presented as definitive evidence of local population decline or direct causal effect.

Despite these limitations, the consistency of the spatial and temporal patterns observed across the monitoring period indicates that the results are ecologically informative. More broadly, the study demonstrates the value of integrating wildlife monitoring, vegetation context, and disturbance mapping within a single analytical framework. Such an approach strengthens environmental impact assessment by improving the ability to distinguish background ecological variability from spatially concentrated disturbance-associated change. In dynamic mining landscapes such as the Ghaap Plateau, this form of long-term, habitat-explicit monitoring provides a practical basis for balancing resource extraction with biodiversity conservation.

## Data Availability

R scripts used for analysis are available from the corresponding author upon reasonable request.

## Appendix

Table 5

**Table 5:** Vegetation units identified at the study site and their characteristics

Attribute	Wild Olive Shrubland	Panveld	Camphor Bush Bushveld	Wild Olive – Camphor Bush Bushveld	Black Thorn Bushveld	Wolhaarkop Sandveld	Rhigozum Grassland	Dwarf Karroid Shrubland
**Location**	Eastern section of active mining area; slopes toward Groenwaterspruit creek.	In and around the mining property (shallow pan depressions).	West of the Panveld, near mining area (mixed shrub/grass flats).	Gently undulating limestone areas and along drainage lines.	Foothills of banded ironstone ridges, NE of mining activity.	Along drainage lines running east–west, below ironstone hills.	Occurs in open flats with few large trees.	Southern border of the main mining property.
**Total Area**	1,741 ha	1880 ha	*Not specified*	*Not specified*	2982 ha	1211 ha	*Not specified*	2721 ha
**Dominant Plant Species**	*Olea europaea* subsp. *africana* (Wild Olive); *Aristida diffusa*; *Eragrostis lehmanniana*.	*Searsia tridactyla*; *Olea europaea* subsp. *africana*; *Senegalia mellifera*.	*Tarchonanthus camphoratus* (Camphor Bush); *Searsia ciliata*; *Diospyros lycioides*; *Ehretia alba*; *Grewia flava*.	*Olea europaea* subsp. *africana*; *Tarchonanthus camphoratus*; *Ziziphus mucronata*; *Boscia albitrunca*.	*Senegalia mellifera* (Black Thorn); *Rhigozum obovatum*; *Boscia albitrunca*; *Boscia foetida*.	*Vachellia erioloba* (Camel Thorn); *Ziziphus mucronata*; *Senegalia mellifera*.	*Vachellia erioloba* (Camel Thorn); *Brachiaria marlothii*; *Enneapogon cenchroides*; *Eragrostis biflora*.	*Pentzia globosa*; *Dicoma capensis*; *Aristida diffusa*; *Eragrostis lehmanniana*.
**Tree and Shrub Layer**	– *Searsia burchellii*, *Senegalia mellifera*, *Boscia albitrunca* (poorly developed)<br > – *Indigofera sessilifolia*, *Asparagus laricinus*, *Melhania rehmannii*, *Lycium cinereum*<br > – Rocky outcrops with succulents (*Aloinopsis orpenii*, *Avonia albissima*, *Euphorbia planiceps*).	– Thickets on pan edges (*Diospyros lycioides*, *Olea europaea*, *Ziziphus mucronata*, *Tarchonanthus camphoratus*)<br > – Dominated by large *Tarchonanthus camphoratus* shrubs<br > – Common shrubs: *Searsia ciliata*, *Diospyros lycioides*, *Ehretia alba*, *Grewia flava*<br > – Trees include *Searsia lancea*, *Ziziphus mucronata*<br > – Sensitive patches of *Rhigozum trichotomum* with endemic species.	– Well-developed dwarf shrub layer: *Pentzia calcarea*, *Chrysocoma ciliata*, *Lycium cinereum*, *Geigeria ornativa*, *Pteronia* spp., *Zygophyllum* spp.<br > – Drainage lines: *Ziziphus mucronata*<br > – Sporadic *Boscia albitrunca*.	– *Senegalia mellifera*, *Rhigozum obovatum*, *Boscia albitrunca*, *Boscia foetida*, *Searsia tridactyla*, *Ehretia alba*<br > – Dwarf shrubs: *Rhigozum trichotomum*, *Barleria alba*, *Asparagus* spp., *Dicoma capensis*, *Hermannia comosa*<br > – Rocky areas: dwarf shrubs (*Rhigozum obovatum*, *Lopholaena cneorifolia*, *Hermannia comosa*, *Waltheria indica*, *Sida ovata*).	– Dominated by large shrubs like *Vachellia hebeclada*, *Tarchonanthus camphoratus*<br > – Smaller shrubs: *Plinthus sericeus*, *Chrysocoma ciliata*, *Lycium cinereum*, *Zygophyllum pubescens*<br > – Sensitive species: *Titanopsis fulleri* (succulent); *Tridentea* spp.; near-endemic *Euphorbia bergii*.	– Woody components: *Grewia flava*, *Senegalia mellifera*, *Ziziphus mucronata* (sparse, with *S. mellifera* dominating where present).	*-*	*-*
**Grass Layer**	Dominated by *Aristida diffusa*, *Eragrostis lehmanniana*, with *Cymbopogon plurinodis* in drainage lines and *Themeda triandra* in deeper soils.	– Grass pans: *Eragrostis* spp.<br > – Shrub pans: *Chrysocoma* spp., *Walafrida* spp., *Pentzia* spp.<br > – Mixed pans: co-dominated by grasses and shrubs.	– Well-developed grass layer dominated by *Aristida congesta*, *Eragrostis lehmanniana*.	– Poorly developed grass layer<br > – Common grasses: *Aristida congesta*, *Eragrostis lehmanniana*.	– Dominated by *Stipagrostis uniplumis*, *Aristida congesta*, *Eragrostis lehmanniana*, *Fingerhuthia africana*<br > – Rocky areas: *Aristida diffusa*, *Heteropogon contortus*, *Panicum maximum*, *Brachiaria nigropedata*, *Eragrostis nindensis*, *Stipagrostis uniplumis*.	– Dominated by *Eragrostis lehmanniana*, *Aristida congesta*, *Stipagrostis uniplumis*, *Schmidtia pappophoroides*, *Cymbopogon plurinodis*<br > – Sensitive species include *Vachellia erioloba*, *Ammocharis coranica*, *Trichodiadema pomeridianum*, *Mestoklema arboriforme* (indicator species).	Dominated by *Brachiaria marlothii*, *Enneapogon cenchroides*, *Eragrostis biflora*, *Eragrostis lehmanniana*, *Stipagrostis uniplumis*.	Dominated by *Aristida diffusa*, *Eragrostis lehmanniana*, *Fingerhuthia africana*, *Themeda triandra*.
**Conservation Considerations**	Rocky outcrops with dwarf succulents (*Aloinopsis orpenii*, *Avonia albissima*, *Euphorbia planiceps*) are high conservation value areas.	Pans are sensitive habitats; classified into grass pans, scrub pans, and mixed scrub/grass pans with variable cover.	*Rhigozum trichotomum* shrub patches are associated with endemic and protected species.	Endemic and protected species are likely present (specialised shrubs and dwarf plants), though none uniquely identified in this unit.	Rocky outcrops harbour endemic/protected species like *Pachypodium succulentum* (considered sensitive).	– Occurrence of trees like *Ficus cordata*, *Boscia foetida*, and protected ferns (*Pellaea calomelanos*).	Protected species include *Vachellia erioloba*, *Ammocharis coranica*, *Trichodiadema* spp. (succulents).	–
